# Laminar Inflammation Responses in the Oligofructose Overload Induced Model of Bovine Laminitis

**DOI:** 10.3389/fvets.2020.00351

**Published:** 2020-07-13

**Authors:** Jiafeng Ding, Shuaichen Li, Lihong Jiang, Yuepeng Li, Xianhao Zhang, Qiaozhi Song, Muhammad A. Hayat, Jian-Tao Zhang, Hongbin Wang

**Affiliations:** ^1^Department of Veterinary Surgery, Northeast Agricultural University, Harbin, China; ^2^Heilongjiang Key Laboratory for Laboratory Animals and Comparative Medicine, Harbin, China

**Keywords:** bovine laminitis, pathogenesis, inflammation, real-time quantitative PCR, oligofructose

## Abstract

Bovine laminitis causes substantial economic losses and animal welfare problems in dairy farms worldwide. Previously published studies have reported that the inflammatory response plays a central role in the pathogenesis of the disease. To our knowledge, inflammation associated with bovine laminitis induced by high levels of exposure to oligofructose (OF) has not been reported and characterized. In fact, the disease manifestations in this model closely approximate those of clinical laminitis. The objective of this study was to characterize the inflammatory response in OF-induced bovine laminitis. A total of 12 Chinese Holstein dairy heifers were utilized in this study. The heifers were randomly divided into two groups, treatment (*n* = 6) and control (*n* = 6). The treatment group heifers were administered OF solutions via a stomach tube (dose: 17 g/kg of body weight). Upon development of a lameness score of 2 with consecutive positive reactions in the same claw, they would be humanely euthanized. Control heifers were administered deionized water (dose: 2 L/100 kg of body weight) and humanely euthanized at 72 h. Real-time quantitative PCR (qPCR) assays were performed to determine the messenger RNA (mRNA) concentrations of inflammatory mediators in the lamellae. Concentrations of interleukin (IL)-1β, IL-6, IL-8, C-X-C motif chemokine ligand-1 (CXCL-1), macrophage cationic peptide-2 (MCP-2), E-selectin, intercellular adhesion molecule-1 (ICAM-1), cyclooxygenase-2 (COX-2), inducible nitric oxide synthase-1 (iNOS-1), and plasminogen activator inhibitor-1 (PAI-1) were significantly increased (*P* < 0.05) in the treatment group. No significant difference was found for tumor necrosis factor alpha (TNF-α), IL-10, CXCL-6, and MCP-1. These results demonstrated and characterized the laminar inflammatory response leading to the pathogenesis of bovine laminitis at the early stages.

## Introduction

Lameness causes substantial economic losses and animal welfare problems in dairy farms worldwide (1, 2). Bovine laminitis is a disease of the foot that results in lameness (3). Afflicted animals present with sole ulcers, sole hemorrhages, and white line disease in dairy cows (4). The prevalence of these lesions is closely related to breeding, management, and environmental factors (5). In recent years, several studies focused on epidemiology (6), pathophysiology (7), histology (8, 9), and proteomics (10) of disease states. However, due to the inconsistencies in clinical cases and the lack of reproducible experimental models, the exact pathogenesis of bovine laminitis remains unclear (11).

In clinical practice, bovine laminitis typically follows systemic inflammatory diseases, including septic pleuropneumonia, metritis, and ruminal acidosis (12, 13). The oligofructose (OF) overload model most closely mimics this clinical presentation, relative to other existing bovine laminitis models. In the OF model, characteristic pathophysiological and histological changes are observed as in natural clinical cases (8, 14). It also has been utilized by several related studies, such as the biomechanics of claw suspensory tissue (15) and the oxidative response of neutrophils to platelet-activating factors (16). Furthermore, this model is a more reliably induced and representative model than others (starch overload and histamine injection models) (17). Over the last two decades, this model has been intensively used in the study of equine laminitis (18–20), but less frequently in bovine laminitis (21).

A clinically similar but distinct disease called sepsis-related laminitis (SRL) has been described in horses (18), which has similar pathophysiological characteristics to systemic inflammatory response syndrome (SIRS) in humans (22). Inflammatory responses play a central role in laminar injury and subsequent failure in the OF-induced equine laminitis, characterized by markedly increases of inflammatory mediator gene expression and leukocyte influx in lamellae tissue (18, 19, 23). These inflammatory mediators primarily contributed to the inflammatory response, including activation of vessel endothelium, adhesion and emigration of leukocytes, and changes in vessel, which resulted in organ injury and failure (24). However, to our knowledge, no existing studies have characterized the inflammatory response and its role in the pathogenesis of OF-induced bovine laminitis. Thus, the laminar messenger RNA (mRNA) expression of multiple cytokines, chemokines, adhesion molecules, and inflammatory molecules was assessed in the context of the OF-induced bovine laminitis model. The objective of this study was to characterize the inflammatory response in a bovine laminitis model as a foundation for future laminitis studies.

## Materials and Methods

### Ethics Statement

The experimental protocols describing the management and care of animals were approved by the Animal Ethics Committee (AEC) of the Northeast Agricultural University (Harbin, China) that monitors compliance with the Animal Welfare Act (2001). All heifers were continuously monitored by the investigators (permission number: SRM-13).

### Animals

A total of 12 Chinese Holstein dairy heifers were used in this study at an average age of 20.67 ± 3.01 months, an average body weight (BW) of 379.71 ± 19.77 kg, and an average body condition score (25) of 3.00 ± 0.23. Each heifer was clinically healthy, had no history of severe systemic diseases, exhibited normal locomotion and posture, and had no claw disorders (solar ulceration, white line disease, etc.). Animals to be enrolled in the study were purchased from the Wandashan Dairy Farm (Harbin, China).

### Model Induction

The claws of heifers were trimmed 10 days prior to initiation of the experiment. For acclimation, heifers were housed in a large animal experimental barn with a concrete floor. Heifers were trained and fed grass hay *ad libitum* (7.5% total water-soluble sugar content). After acclimation, all heifers could accept clinical examination without any discomfort, could be led to walk and trot by hand, and agreed to lift the distal front limb for foot palpation and hoof testing. The heifers were randomly arranged into two groups, including a treatment group (*n* = 6) and a control group (*n* = 6). Each animal in the treatment group was administered OF solutions (Bailong Biotech, Inc., Dezhou, China; purity, 98%; dose, 17 g/kg BW in 2 L/100 kg of BW warm deionized water) into the rumen via gastric tube, and control group heifers were given 2 L/100 kg of BW warm deionized water by the same method, as previously described (14, 17). A 5% OF dose was administered twice daily before the experiment for 3 days.

Orthopedic examinations were performed (14), including locomotion assessment, hoof testing, and weight shifting at −24, 0, 6, 12, 18, 24, 36, 48, 60, and 72 h. In the locomotion assessment, the heifers were led by hand to walk and trot in a straight line and to turn in a small circle on the same surface. Five licensed veterinarians assessed the locomotion scores of each heifer according to previous study (Table 1) in an experimentally blinded manner (26). Heifers receiving a score of ≥2 by all veterinarians were considered to be lame. In hoof testing, the front legs were lifted up, while a hoof tester was applied over the site of the axial sole–bulb junction and the central region of the dorso-abaxial claw wall in all front claws. A suitable pressure was applied to assess fasciculation in the musculus triceps. Animals were scored based on their attempts to withdraw their legs. Reactions to hoof testing were subjectively classified as none, slight, or marked. In weight shifting, heifers were observed whether they shifted their weight to another side during the examination period.

**Table 1:** Locomotion scoring system adapted from Sprecher et al. (26).

**Lameness score**	**Description**	**Assessment criteria**
1	Normal	The heifer stands and walks with a level-back posture. Its gait is normal.
2	Mildly lame	The heifer stands with level-back posture but walks with an arched-back posture. Its gait is normal.
3	Moderately lame	The heifer stands and walks with an arched-back posture. Its gait develops a short-striding step with 1 or more limbs.
4	Lame	The heifer stands and walks with an evident arched-back posture. Its gait develops a deliberate step at a time. The heifer favors 1 or more limbs/feet.
5	Severely lame	The heifer demonstrates an inability or extreme reluctance to bear weight on 1 or more of its limbs/feet.

For the consideration of animal welfare, supportive therapy was provided (14). Ringer's acetate (Heping Animal Medicine, Inc., Harbin, China; dose, 15 ml/kg of BW) and sodium bicarbonate (Heping Animal Medicine, Inc., Harbin, China; specification, 84 g/L; dose, 1.5 ml/kg of BW) were administered at 18 and 24 h, and calcium borogluconate (Heping Animal Medicine, Inc., Harbin, China; specification, 14 mg of Ca/ml; dose, 1.4 ml/kg of BW) was administered by jugular infusion at 18 h.

### Sample Acquisition

When the treatment group heifers were scored ≥2 and had consecutive positive reactions in the same claw (14, 17), the animals were humanely euthanized. The control heifers were humanely euthanized at 72 h. The front limbs were rapidly removed by disarticulation of the metacarpophalangeal joint. Several tissues (approximately 2.5–3.0 cm^2^) were excised using a band saw, consisting of the third phalanx, lamellae tissue, and the central part of the dorso-abaxial claw wall (27, 28). Lamellae tissue (~1 cm^2^) was rapidly dissected from the larger tissue blocks using a lancet. Several dissected lamellae were immediately snap frozen in liquid nitrogen and later stored at −80°C. Remaining lamellae samples were fixed in 10% neutral buffered formalin (Kanning Medicine, Inc., Zhongshan, China) for at least 24 h. The formalin-fixed lamellae was treated with graded alcohol, xylene, and paraffin using an automatic tissue-processing machine (8). Sections (4 μm thick) were cut and stained with Mayers hematoxylin and eosin (H&E) for observation of histological changes within the epidermal and dermal lamellae and periodic acid–Schiff (PAS) staining for basement membrane changes. Veterinary pathologist Prof. Guangxing Li (Northeast Agricultural University) and medical pathologist Prof. Zhao (Harbin Medical University) blindly read the sections in a random order.

### RNA Isolation and cDNA Synthesis

Total RNA was extracted from three separate lamellae samples of 12 heifers using Absolutely RNA Miniprep kits (Stratagende, Inc., California, USA). The quantity and purity of extracted RNA were measured using a NanoDrop^TM^ One Microvolume spectrophotometry (Thermo Scientific, Massachusetts, USA). The integrity of RNA was determined by 1% agarose gel electrophoresis (Bio-Rad Laboratories, California, USA). One microgram of total RNA was reverse transcribed using the PrimeScript^TM^ RT reagent kit (Takara, Dalian, China) with genomic DNA (gDNA) eraser according to the manufacturer's instructions. Subsequently, complementary DNA (cDNA) was diluted with DNase/RNase free water (dilution rate, 1:4) (Takara, Dalian, China) and stored at −20°C.

### Real-Time Quantitative PCR

The quantitative PCR (qPCR) was conducted using a LightClycler 480 (Roche, Indiana, Germany), and quantification was assessed with SYBR Premix Ex Taq^TM^ II (Takara, Dalian, China). The primers for interleukin (IL)-1β, IL-6, IL-8, IL-10, tumor necrosis factor alpha (TNF-α), C-X-C motif chemokine ligand (CXCL)-1, CXCL-6, macrophage cationic peptide (MCP)-1, MCP-2, E-selectin, intercellular adhesion molecule-1 (ICAM-1), cyclooxygenase-2 (COX-2), inducible nitric oxide synthase-1 (iNOS-1), plasminogen activator inhibitor-1 (PAI-1), and housekeeping genes (β-actin, GAPDH, and UXT) used in this study were the same as previously reported (27–31) and were designed to target bovine-specific sequences (Table 2). The specificity of selected primer sequences was verified using the Basic Local Alignment Search Tool (BLAST) from the National Center for Biotechnology information (NCBI) database (http://blast.ncbi.nlm.nih.gov/). A 20-μl PCR mixture contained 2 μl sample cDNA and 18 μl PCR master mix. The master mix contained 10 μl SYBR green dye, 1.6 μl each primer solution (forward and reverse primers, each 10 μM), and 6.4 μl DNase/RNase free water. The PCR conditions were as follows: 95°C for 1 min (ramp rate, 4.4°C/s); 40 cycles of amplification (quantification analysis model), 95°C for 5 s (ramp rate, 4.4 °C/s) and 60°C for 1 min (ramp rate, 2.2°C/s); 1 cycle of melting (melting curves analysis model), 95°C for 5 s (ramp rate, 4.4°C/s), 60°C for 1 min (ramp rate, 2.2°C/s), increasing to 95°C (ramp rate, 0.11°C/s); 1 cycle of cooling, 50°C for 30 s (ramp rate, 2.2°C/s). All amplified cDNA fragments were confirmed by gel electrophoresis and melting curve analysis. The values of cycle threshold crossing (Ct) were calculated by the Light Cycler 480 software (version 1.5.0, Roche, Germany). Template DNA (10-folded serial dilution) were used to generate standard curves and calculate the efficiency for each PCR reaction. Negative controls consisted of DNase/RNase free water, and each sample was analyzed in triplicate.

**Table 2:** Gene, sequence, amplification size, and efficiency of the primers used for quantitative PCR (qPCR).

**Gene**	**Polarity**	**Sequence (5^′^ → 3^′^)**	**Size (bp)**	**Amplification efficiency (%)**	**NCBI accession no**.
**Cytokine**					
IL-1β	Forward	ATTCTCTCCAGCCAACCTTCATT	100	96	NM_174093
	Reverse	TTCTCGTCACTGTAGTAAGCCATCA			
IL-6	Forward	ATGACTTCTGCTTTCCCTACCC	180	105	NM_173923
	Reverse	GCTGCTTTCACACTCATCATTC			
IL-8	Forward	GACAGCAGAGCTCACAAGCATCT	105	94	NM_173925.2
	Reverse	AAGCTGCCAAGAGAGCAACAG			
IL-10	Forward	ACAGGCTGAGAACCACGGGC	175	90	NM_174088.1
	Reverse	GACACCCCTCTCTTGGAGCTCACT			
TNF-α	Forward	CCAGAGGGAAGAGCAGTCCC	114	92	NM_173966.3
	Reverse	TCGGCTACAACGTGGGCTAC			
**Chemokine**					
CXCL-1	Forward	CATCCAGAGCGTGAAGGTGA	100	97	NM_175700.2
	Reverse	GGTGGGGTTGAGACACACTT			
CXCL-6	Forward	TGAGAGAGCTGCGTTGTGTG	119	103	NM_174300.2
	Reverse	GGTGGCTATCACTTCCACCT			
MCP-1	Forward	GCAATTAACTCCCAAGTCGCC	161	95	NM_174006.2
	Reverse	TGCTTGGGGTCTGCACATAA			
MCP-2	Forward	ATCACCAACAGCCAGTGTCC	133	91	NM_174007.1
	Reverse	TCGGTGTTCGGGACTTTTGG			
**Adhesion molecule**					
ICAM-1	Forward	CGACCACAGGAGCAACTTCT	171	106	NM_174348.2
	Reverse	TCGCACTTCAGGGTCTGTTC			
E-selectin	Forward	CATCCTCAGAACGGCACTGT	167	97	NM_174181.2
	Reverse	ACTTCACAAACTGGGACCCG			
**Inflammatory molecule**					
COX-2	Forward	ATCTACCCGCCTCATGTTCCT	187	93	AF031698
	Reverse	GGATTAGCCTGCTTGTCTGGA			
iNOS	Forward	CAGGATGACCCCAAACGTCA	190	101	XM_024979646.1
	Reverse	CCTTCTGGTGAAGCGTGTCT			
PAI-1	Forward	TCTTCCACAAGTCCGATGGC	142	92	XM_024984644.1
	Reverse	ATGCTGAGAGTGTTCCCGTG			
**Housekeeping gene**					
ACTB	Forward	ACTTGCGCAGAAAACGAGAT	123	97	BT030480
	Reverse	CACCTTCACCGTTCCAGTTT			
GAPDH	Forward	GGGTCATCATCTCTGCACCT	176	95	DQ402990
	Reverse	GGTCATAAGTCCCTCCACGA			
UXT	Forward	TGTGGCCCTTGGATATGGTT	101	101	BC108205.1
	Reverse	GGTTGTCGCTGAGCTCTGTG			

### Data Analysis

The 2^−ΔΔ*Ct*^ method was used to calculate changes in relative gene expression. Data analysis was performed using GraphPad Prism (version 7.04, GraphPad Software, Inc., San Diego, USA). The data fulfilled the assumption of a Gaussian distribution, according to a Shapiro–Wilk normality test. The data were analyzed by Student's *t*-test. *P* < 0.05 was considered significant. All data are presented as mean ± standard deviation (SD).

## Results

### Clinical Data

Heifers of the treatment group developed depression, anorexia, watery diarrhea, tachycardia, tachypnea, and pyrexia. The signs of depression, anorexia, and watery diarrhea were observed at 6–12 h, and severe signs occurred at 24–36 h, then gradually recovered. Generally, persistent tachycardia, hypopnea, and pyrexia could be observed from 6 to 72 h and became increasingly severe before 36 h, then gradually recovered. At 36 h, the rate of heart beat reached the maximum mean value (69.83 ± 13.82 beats/min), rectal temperatures also reached the maximum mean value (39.25 ± 0.40°C), and respiratory rate reached the minimum mean value (15.00 ± 1.90 counts/min). These heifers were otherwise graded as normal at −24 and 0 h. The signs of lameness were observed at 36 h. At 72 h, the locomotion scores of them were ≥2. From 36 to 60 h, a variable clinical observation for hoof testing examination was observed in the animals. At 72 h, each of them had a consecutive reaction in the same claw. All heifers developed weight shifting involving both front and hind legs. Weight shifting was first observed in two heifers at 12 h and later in all remaining heifers between 36 and 72 h.

Heifers in the control group failed to develop clinical signs of systemic inflammatory disease. During the assay period, clinical observations were normal, for heart rate (48.92 ± 1.70 beats/min), rectal temperature (38.31 ± 0.22°C), and respiratory rate (23.35 ± 1.11 counts/min). They were graded as normal (locomotion score = 1) and did not exhibit pain reactions during hoof testing examination with no signs of weight shifting.

### Laminar Histopathology of Claws

**Figure 1:**
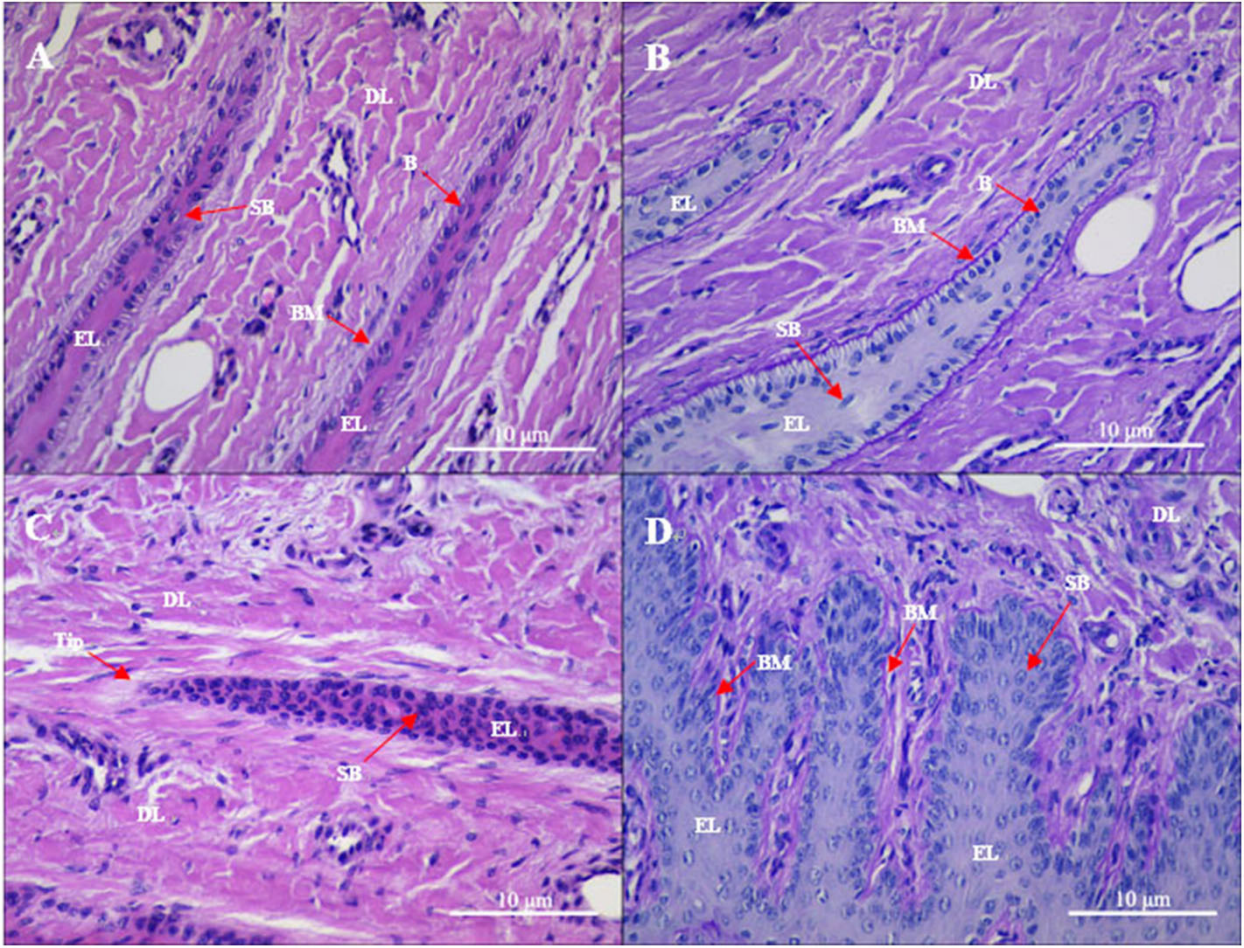
Gross sections of the lamellae layer from **(A,B)** control and **(C,D)** oligofructose treated heifers. Labels in the sections: B, basal cells; SB, surprabasal cells; BM, basement membrane; DL, dermal lamellae; EL, epidermal lamellae; WBC, white blood cells; Tip, the tip of the epidermal lamellae). **(A)** H&E stain. The normal appearance of the dermo-epidermal junction consists of numerous interlocking lamellae. **(B)** Periodic acid–Schiff (PAS) stain. The tip of the epidermal lamellae is rounded and in a close association with the basement membrane. **(C)** H&E stain. Stretched epidermal lamellae and increased numbers of suprabasal cells are observed. **(D)** PAS stain. The basement membrane has an attenuated appearance; the basal cells are enlarged and wider in the dermal lamellae.

Gross observation of the control group revealed normal lamellae. From histological sections, the epidermal and dermal layers of the lamellae were intimately connected (Figures 1A,B). In the PAS-stained sections, the epidermal lamellae had a rounded tip and was closely connected to the basement membrane. The basement membrane appeared as a consecutive and distinct line. In the HE-stained sections, the basophilic nuclei of basal cells were oval and located away from the basement membrane with regular nuclear polarity shifts. Suprabasal cells were almost parallel to the direction of epidermal lamellae. Hyperemia, hemorrhage, and a few inflammatory cells (mainly lymphocytes and macrophages) were observed, albeit rarely, in the dermal lamellae.

Gross observation of the treatment group revealed a patchy distribution of dermal hyperemia in the dorsal and bottom region of the lamellae. In the histological sections, the epidermal lamellae appeared stretched and with a pointed tip (Figures 1C,D). In the PAS-stained sections, the basement membrane had a folded and attenuated appearance, and occasionally separated from the basal cells. Basal cells had rounded and centrally positioned nuclei. Nuclei had a coarse chromatin network. Suprabasal cells appeared hyperplastic. Hyperemia, hemorrhage, and pronounced inflammatory infiltrates (granulocytes and monocytes) were observed in the dermal lamellae.

### Laminar Inflammatory Cytokine mRNA Concentration

**Figure 2:**
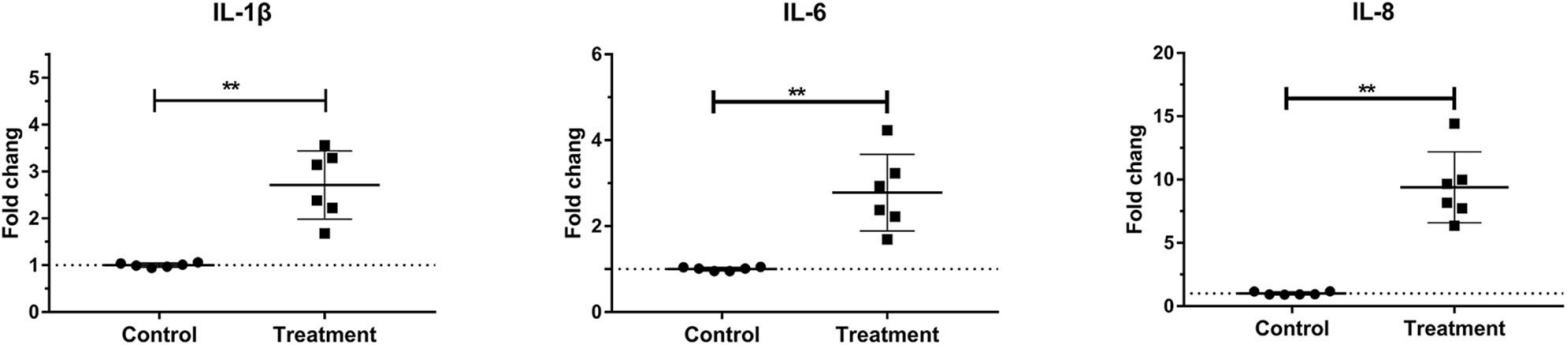
Mean fold changes in interleukin (IL)-1β, IL-6, and IL-10 messenger RNA (mRNA) expression after oligofructose administration in heifers. Significant increases in cytokines relative to the control group are indicated as **P < 0.01.

Laminar mRNA concentrations of the cytokines IL-1β, IL-6, and IL-8 were significantly increased in the treatment group (*P* < 0.01) relative to the control group. There was no change in laminar mRNA expression of IL-10 and TNF-α (Table 3 and Figure 2).

**Table 3:** Fold changes in laminar gene expression of inflammation mediators after oligofructose administration.

**Inflammation mediator**	**Fold change**	***P* value**
**Cytokine**		
IL-1β	2.76 ↑	0.0022
IL-6	2.63 ↑	0.0045
IL-8	9.53 ↑	0.0007
IL-10	0.61	0.0694
TNF-α	0.84	0.9879
**Chemokine**		
CXCL-1	5.04 ↑	0.0001
CXCL-6	0.92	0.6314
MCP-1	0.93	0.2987
MCP-2	3.05 ↑	0.0004
**Adhesion molecule**		
ICAM-1	1.81 ↑	0.0478
E-selectin	2.90 ↑	0.0381
**Inflammatory molecule**		
COX-2	1.30 ↑	0.0259
iNOS	5.13 ↑	0.0225
PAI-1	1.43 ↑	0.0480

*Values displayed are mean fold change from control values*.

*NS, nonsignificant changed values; ↑, significant increased values*.

### Laminar Chemokine mRNA Concentration

**Figure 3:**
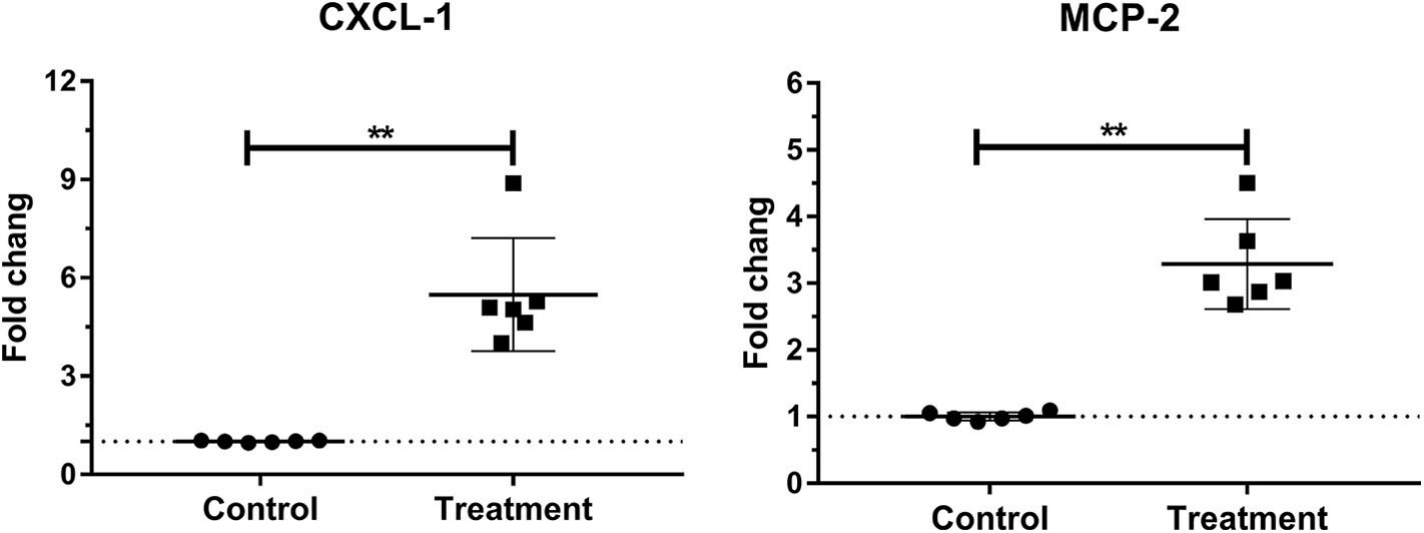
Mean fold changes in CXCL-1 and MCP-2 messenger RNA (mRNA) expression following oligofructose administration in heifers. Significant increases in chemokines relative to the control group are indicated as **P < 0.01.

Laminar mRNA concentrations of chemokine CXCL-1 and MCP-2 were significantly increased in the treatment group (*P* < 0.01) compared to the control group. There was no change in laminar mRNA expression of chemokine CXCL-6 and MCP-1 (Table 3 and Figure 3).

### Laminar mRNA Concentration of Endothelial Adhesion Molecule

**Figure 4:**
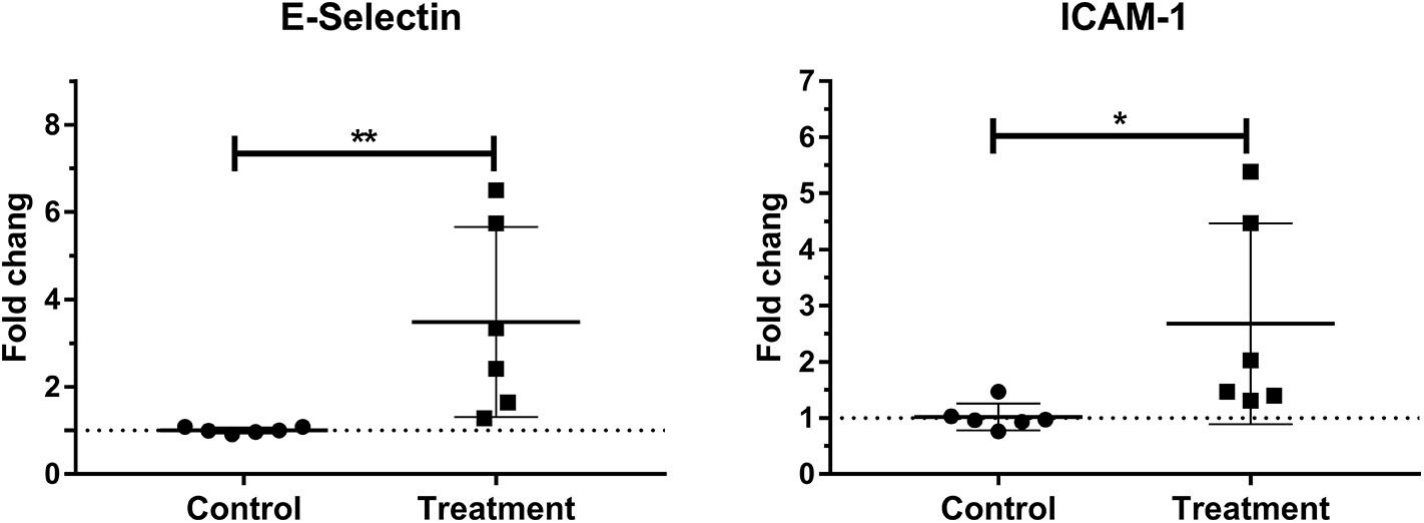
Median fold changes in E-selectin and intercellular adhesion molecule-1 (ICAM-1) messenger RNA (mRNA) expression after oligofructose administration in heifers. Significant increases in adhesion molecules relative to the control group are indicated as *P < 0.05, **P < 0.01.

Laminar mRNA expressions of the endothelial adhesion molecule E-selectin (*P* < 0.01) and ICAM-1 (*P* < 0.05) were significantly increased in the treatment group relative to the control group (Table 3 and Figure 4).

### Laminar mRNA Concentration of Inflammatory Molecule

**Figure 5:**
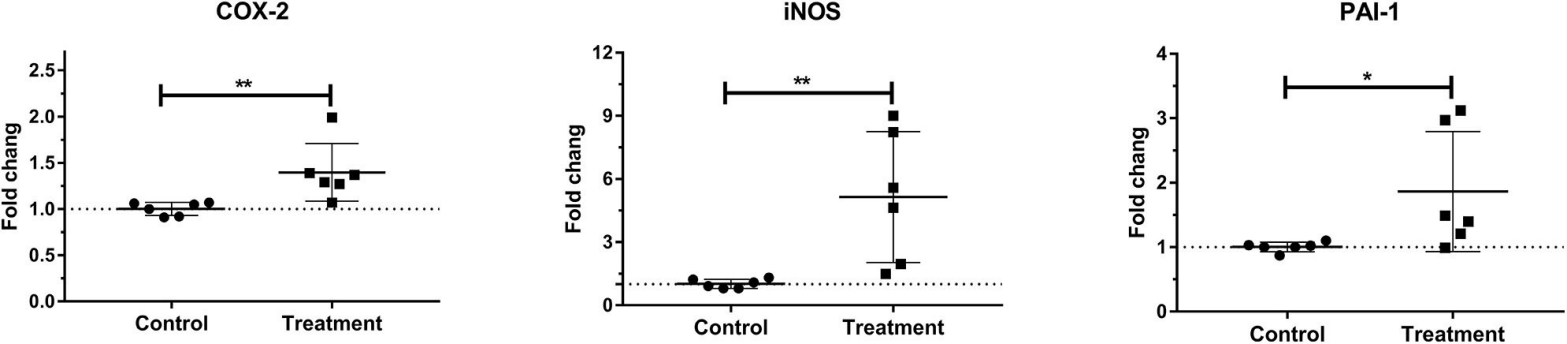
Mean fold changes in cyclooxygenase-2 (COX-2), inducible nitric oxide synthase-1 (iNOS-1), and plasminogen activator inhibitor-1 (PAI-1) messenger RNA (mRNA) expression after oligofructose administration in heifers. Significant increases in inflammatory molecules relative to the control group are indicated as *P < 0.05, **P < 0.01.

Laminar mRNA expression of inflammatory molecules COX-2 (*P* < 0.01), iNOS (*P* < 0.01), and PAI-1 (*P* < 0.05) was significantly increased in the treatment group when compared to the control group (Table 3 and Figure 5).

## Discussion

Bovine laminitis is also referred to as diffuse aseptic pododermatitis, which results in local and systemic clinical signs (32). It is characterized by claw pain and lameness (33). In clinical practices, laminitis is typically secondary to severe systemic inflammatory disease (e.g., septic pleuropneumonia, metritis, ruminal acidosis, etc.).

In the present study, the OF overload model was employed to induce a bovine model of laminitis. This model was selected for the following reasons: (1) the histamine injection model and the starch overload model are not reliable; (2) the histological changes in the OF model are similar to those observed in clinical laminitis cases; and (3) the OF model has been widely used in the studies of bovine and equine laminitis.

The laminitis-inducing mechanism of OF is similar to other carbohydrate overload models. A high intake of nonstructural carbohydrates, which could reduce the rumen pH, results in the death and lysis of Gram-negative bacteria. This bacterial die-off compromises the intestinal barrier functions. This allows lipopolysaccharide (LPS) to translocate from the digestive tract into the peripheral circulation (17, 34, 35). Subsequently, free LPS activates systemic inflammatory responses and leads to multiorgan damage, such as rumenitis (36), synovitis (37), and liver impairment (38).

In the present study, heifers in the treatment group developed characteristic signs of bovine laminitis, and histological changes of their lamellae tissues were consistent with clinical laminitis cases. Those results corroborated those of previous studies, which indicated the successful induction of bovine laminitis (14, 17). This study reported that laminar mRNA expressions of several inflammatory cytokines, chemokines, endothelial adhesion molecules, and inflammatory molecules were increased at the early stage of the OF-induced bovine laminitis. To our knowledge, this is the first study to characterize the inflammatory response in this model. Our findings were similar to equine SRL and human SIRS/sepsis (16, 39), indicating that lamellae failure might be caused by a systemic inflammatory response, as is the case with organ failure in human SIRS/sepsis.

Pathogen-associated molecular pattern molecules (PAMPs) (e.g., LPS) activate leukocytes and other innate immune cells (40, 41). Inflammatory mediators including inflammatory cytokines, chemokines, endothelial adhesion molecules, and inflammatory molecules could be produced by PAMP-activated cells (24). Meanwhile, the activated leukocytes pass through the activated endothelium and migrate into the tissues of target organs (42). Activated tissue macrophages, migratory leukocytes, and multiple inflammatory mediators together constitute the inflammatory response, leading to organ injury and failure resulting from sepsis (43).

In this study, gene expression of inflammatory cytokines IL-1β, IL-6, and IL-8 was increased in the lamellae of the treatment group. Previous studies reported that the laminar expression of IL-1β, IL-6, and IL-8 was related to the degree of lamellar injury (44). Furthermore, these inflammatory cytokines are important components of the innate immune response, which have also been reported to participate in human SIRS/sepsis (22). A previous study found that the increased plasma level of IL-1β was negatively correlated with the progression of organ dysfunction and mortality in septic patients (45). Another study reported that IL-1β commonly underwent an early but transient burst of expression after being exposed to the stimulation of endotoxin (46). Similarly, an increase in IL-1β at the early stage of the development of laminitis was observed here, indicating that it might take part in the productions of other cytokines.

In human sepsis, IL-6 expression was correlated with organ injury and apoptosis (47). Recently, a commercial IL-6 signaling-related drug was used as a potential therapeutic for the treatment of rheumatoid arthritis and sepsis (48, 49). It has been reported that IL-6 signaling-related glycoprotein 130 (gp130) might play an important role in the stretching of epidermal lamellae and separation from the dermal lamellae (50). These changes are also histologically characteristic of clinical laminitis cases, which were also observed in this study. It has been reported that IL-8 primarily acts as a chemokine, promoting migration of neutrophils into the lamellae in OF-induced bovine laminitis (51). It has also been reported to be expressed by endothelial cells following exposure to PAMPs and TNF-α (46).

Tumor necrosis factor-α is a prominent inflammatory cytokine during human SIRS/sepsis. However, a notable lack of TNF-α expression was observed in this study. In OF-induced equine laminitis, the expression of TNF-α was also not increased early on in the lamellae, but the serum concentration of TNF-α was significantly increased (52). These observations are indicative of a systemic cytokine response. An early transient burst of TNF-α occurring in this process may be another possible explanation. A lung sepsis model in baboon reported that the gene expression of TNF-α was also not increased, but that of IL-1β and IL-6 was increased in the target organ (53). Similar observations were made here.

Like TNF-α, IL-10 expression was not increased in this study, indicating that the lamellae did not mount an anti-inflammatory response during the early stage of laminitis. It is well established that IL-10 is an anti-inflammatory cytokine that serves to temper immune responses (42). Increased IL-10 has generally been associated with improved prognostic outcomes in septic patients (54). The expression of IL-10 was not increased, which can be explained by the lack of TNF-α response in this study (18). This speculation is based on previous studies, in which the plasma concentration of IL-10 was decreased by the administration of antimonoclonal TNF-α antibodies in chimpanzees with sepsis (55).

The expression of the chemokines CXCL-1 and MCP-2 were increased in the lamellae, suggesting that the migration of neutrophils and mononuclear cells into lamellae maybe involved in this inflammatory response. In OF-induced equine laminitis, increased gene expression of CXCL-1 and MCP-2 and the migration of neutrophils and mononuclear cells into lamellae are observed (20). A recent study reported that CXCL-1 could induce neutrophils to infiltrate the sites of bacterial infection and was involved in the granulopoiesis and mobilization of neutrophils in sepsis (56). Furthermore, MCP-2 is another important chemokine in inflammation and immunomodulation, which could activate mononuclear cells and direct them into target tissues (57).

The expressions of the adhesion molecules E-selectin and ICAM-1 were increased in lamellae, indicating that endothelial activation occurred in this study. The increased expression of adhesion molecules is a characteristic of endothelial activation, which leads to the adhesion of circulating leukocytes. The expression of E-selectin and ICAM-1 could be explained by the upregulated expression of IL-1β and IL-6, as these proinflammatory cytokines positively induce the transcriptional regulation of E-selectin and ICAM-1 (58).

In this study, the expressions of the inflammatory molecules COX-2, iNOS, and PAI-1 were increased in the lamellae. Similar results were reported in OF-induced equine laminitis, indicating that these inflammatory molecules might play similar roles in the two laminitis models (18). COX-2 is known as a crucial mediator in the inflammatory response, as the various physiological effects of numerous prostanoids (the products downstream of COX-2), including platelet aggregation, vasomotor, and proinflammatory effects (59). As such, COX-2 is a therapeutic target for nonsteroidal anti-inflammatory drugs (NSAIDs). The increased expression of COX-2 could be speculated from the increased proinflammatory cytokine IL-1 in this study, as the expression of COX-2 was mainly driven by the activation of nuclear factor-kappa B (downstream of proinflammatory cytokine signaling) (60). However, the pathological functions of COX-2 in bovine laminitis were still unclear. In equine laminitis, laminar COX-2 expression was observed in multiple laminitis-related cells (61), especially laminar basal epithelial cells (LBEC). Furthermore, LBEC are responsible for maintaining the integrity of the epidermal lamellae and basement membrane. Therefore, it is possible that COX-2 maybe involved in the failure of the lamellae and that COX-2 may serve as a prognostic indicator of laminitis. In endotoxin-induced sepsis, COX-2-deficient mice exhibited increased survival and decreased leukocyte infiltration into kidneys and lungs, when compared to wild-type mice (62).

Expression of the vasoactive substances iNOS and PAI-1 were increased in lamellae. Overexpression of iNOS results in excessive nitric oxide (NO), which causes systemic/local hypotension and organ dysfunction in sepsis (63). Elevated PAI-1 expression was reported to be a crucial regulator of fibrinolysis by inhibiting plasminogen activator, which could lead to disseminated intravascular coagulation and organ dysfunction in septic patients (64). Intravascular coagulation has been observed in bovine lamellae with laminitis (9). Recently, PAI-1 was determined to be a significant predictor of severity through a meta-analysis of human sepsis cases (65).

In conclusion, this study reported the occurrence of laminar inflammatory responses at the early stages of OF-induced bovine laminitis. Expression of multiple mediators of inflammation increased, indicating that the inflammatory injury might play an important role in the pathogenesis of laminitis. Moving forward, more in-depth molecular studies on inflammatory signaling pathways [e.g., nuclear factor kappa B (NF-κB) mitogen-activated protein kinase (MAPK) pathways] are needed to discover effective anti-inflammatory agents for the treatment of bovine laminitis.

## Data Availability Statement

The raw data supporting the conclusions of this article will be made available by the authors, without undue reservation, to any qualified researcher.

## Ethics Statement

The animal study was reviewed and approved by Animal Ethics Committee (AEC) of Northeast Agricultural University, Harbin, China (permission number: SRM-13).

## Author Contributions

JD designed and performed the experiments, analyzed data, and wrote the manuscript. SL, LJ, YL, XZ, QS, and MH performed the animal experiments and contributed to the technical discussions. J-TZ and HW designed the experiments and provided guidance. All authors contributed to the article and approved the submitted version.

## Conflict of Interest

The authors declare that the research was conducted in the absence of any commercial or financial relationships that could be construed as a potential conflict of interest.

## Supplementary Material

Video S1 The treatment group heifers became lamed after oligofructose overload administration.

Video S2 The treatment group heifers refused to move after oligofructose overload administration, although researchers led them with a big strength.

Figure S1 The hoof band of heifers became red and swell after oligofructose overload administration.
