# Supplementary figures and images for: Laminar Inflammation Responses in the Oligofructose Overload Induced Model of Bovine Laminitis
Source: Front Vet Sci. 2020 Jul 13;7:351. doi: 10.3389/fvets.2020.00351 (PMC7381234; doi:10.3389/fvets.2020.00351)

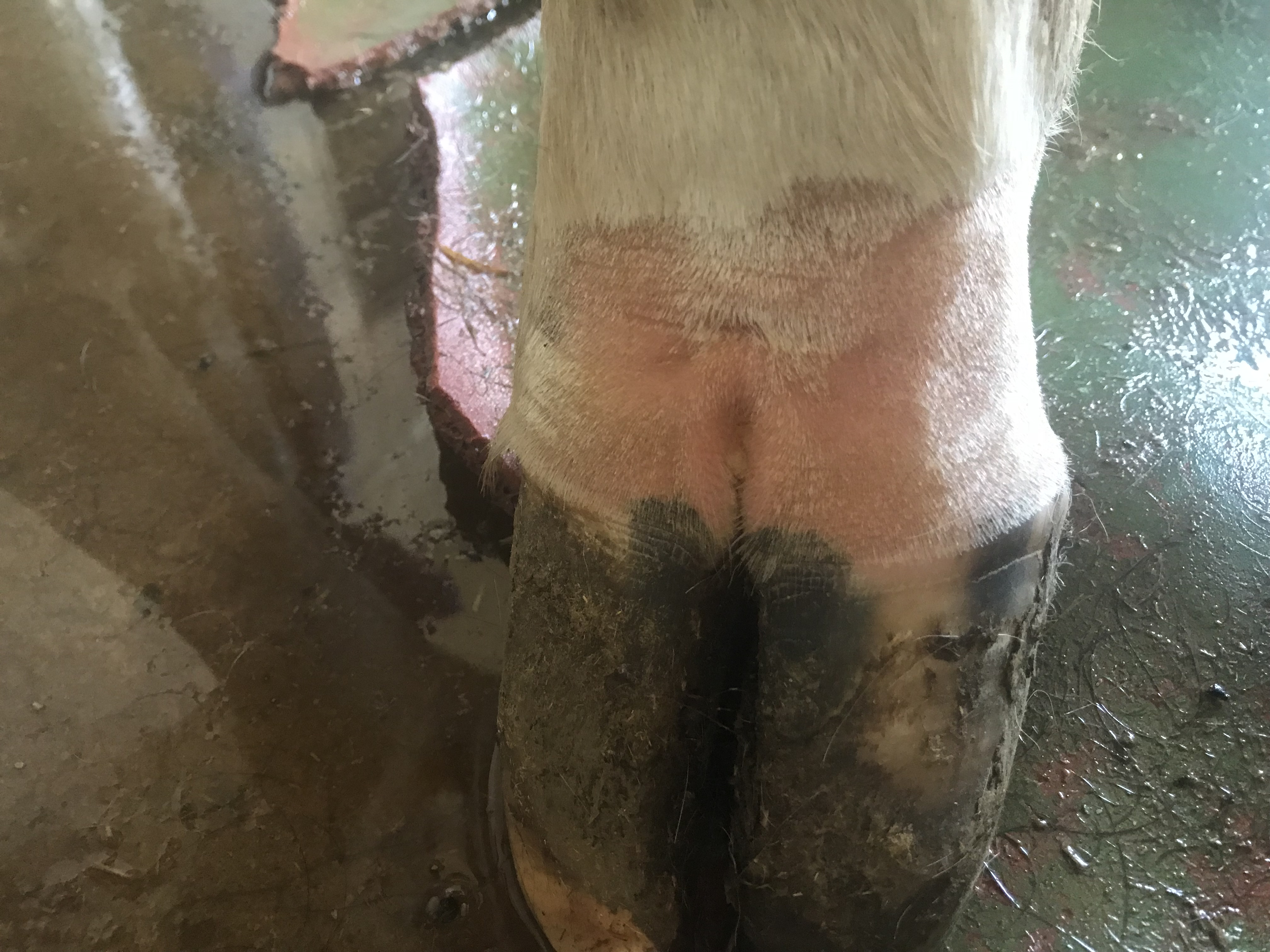

Supplement: Figure S1 — The hoof band of heifers became red and swell after oligofructose overload administration. [file Image_1.JPEG]
